# Molecular Characterization of Culturable Yeasts and Nonspore-Forming Bacteria Associated With Fermented Kapok Seeds (*Kantong*), a Traditional Food Condiment in Ghana

**DOI:** 10.1155/ijfo/6452183

**Published:** 2025-06-23

**Authors:** Elmer Nayra Ametefe, Line Thorsen, Harry Danwonno, Righteous Kwaku Agoha, Richard L. K. Glover, Victoria Pearl Dzogbefia, Lene Jespersen

**Affiliations:** ^1^Department of Biochemistry, Cell and Molecular Biology, University of Ghana, Accra, Greater Accra Region, Ghana; ^2^BACTHERA A/S Copenhagen, Copenhagen, Denmark; ^3^African Science, Technology and Policy Institute (ASTePI), Pretoria, South Africa; ^4^Department of Biochemistry and Biotechnology, Kwame Nkrumah University of Science and Technology, Kumasi, Ashanti Region, Ghana; ^5^Department of Food Science, University of Copenhagen, Frederiksberg, Copenhagen, Denmark

**Keywords:** fermented foods, kantong, microbial ecology, probiotic reservoir

## Abstract

Fermented kapok seeds, known as *kantong* in northern Ghana, serve as a traditional food condiment which provides flavor and improves the protein content of soups. In this study, the occurrence of yeasts, lactic acid bacteria (LAB), and other nonspore-forming bacteria in *kantong* was investigated. Microbial enumeration and phenotypic characterizations on isolated strains were performed. Molecular methods were also employed for grouping and identification of strains, and these included random amplification of polymorphic DNA (RAPD) using *Escherichia coli* phage–derived M13 primer (M13-PCR typing), repetitive element PCR typing (rep-PCR), and 16S rRNA gene sequencing. After a 48-h fermentation period, microbial load ranged from 4.77 ± 0.11 to 8.9 ± 0.1 log_10_ CFU/g. The pH of the fermenting condiment decreased from 6.5 to 4.7 during the fermentation period. A total of 190 LAB, 53 enterobacteria, and 39 yeasts were identified at species levels using both phenotypic and molecular methods. The LAB included *Pediococcus acidilactici*, *Weissella paramesenteroides*, *Pediococcus pentosaceus*, *Weissella confusa*, and *Lactiplantibacillus plantarum*; the enterobacteria isolated were *Acinetobacter baumannii*, *Klebsiella pneumoniae*, *Enterococcus faecium*, *Escherichia coli*, and *Enterobacter cloacae*; and the yeasts identified were *Nakaseomyces glabratus*, *Cyberlindnera fabianii*, *Pichia kudriavzevii*, and *Saccharomyces cerevisiae*. This work presents fermented kapok seeds as a reservoir of microorganisms, some of which could possess some technological properties which could be harnessed to enhance the nutritional value of Ghanaian foods as well as improve gut health as probiotics. It also reveals the presence of enterobacteria in this spontaneous fermentation, thus impacting the safety of the product and the need for starter culture development.

## 1. Introduction

Fermentation of foods in West Africa is an age-old craft usually carried out by the elderly women in the region and handed down from one generation to another [1, 2]. It plays a significant role in enhancing the taste, flavor, and nutritional value as well as shelf life of foods in this region [3, 4]. Food items that undergo fermentation may include cereals, oilseeds, vegetables, milk, meats, and fish. Although many of these traditional fermented foods are a result of spontaneous fermentations resulting in an undefined microbial ecology and uncertain by-products or toxins [4, 5], they are also a reservoir of a plethora of microorganisms with potential technological properties with implications for food processing as well as the consumer gut health [6, 7].

Fermented seed condiments play a vital role in the traditional diets of many West African communities, enhancing the flavor of various soups and sauces while supplying essential proteins and vitamins [8]. In Northern Ghana, for example, the Dagomba tribe primarily produces and consumes *kantong*, a fermented seed condiment made from *Ceiba pentandra* (kapok seeds). This condiment serves as an economical protein source in the region [9]. Despite its regional importance, few studies have characterized the microbial succession underpinning kantong fermentation, leaving a critical gap in our understanding of its safety, consistency, and sensory qualities. The preparation of *kantong* involves several steps: first, the kapok seeds are cleaned by winnowing and washing to remove light debris such as fibers, husks, and stones. Next, the cleaned seeds are sun-dried for a period that depends on weather conditions. Once sufficiently dried, the seeds are dehulled by pounding in a mortar, followed by sifting. The resulting fine flour is mixed with cassava flour in a 10:1 ratio of seed flour to cassava flour. Water is added to form a thick paste, which is fermented over 48 h. During the first 24 h, the paste is covered in a bowl; it is then uncovered, manually mixed, and exposed to sunlight for about 8 h to allow the surrounding liquid to evaporate, before being covered again for the rest of the fermentation period. After 48 h, the paste is shaped into small pellets and dried. These dried pellets (dps) are then kneaded by pounding in a mortar with a slight sprinkling of water. The kneaded product is molded into small balls, ready for sale and consumption [10].

Spontaneously fermented West African seed condiments, such dawadawa (*Parkia biglobosa*), maari (*Adansonia digitata*), and bikalga (*Hibiscus sabdariffa*), have been investigated [11, 12] and observed to harbor several species of bacteria, yeasts, and molds [11–14]. Spontaneous fermentation of these seed condiments commences with raw seeds that harbor a diverse array of epiphytic and soil microbiota, including *Micrococcus*, *Corynebacterium*, and low concentrations of dormant *Bacillus* spores, which act as the inoculum for fermentation [15]. Some fermented seed condiments are boiled to soften them and reduce the vegetative load, resulting in the inactivation of most Gram-negative bacteria and nonspore-forming organisms, while heat-resistant *Bacillus* spores, notably *Bacillus subtilis*, *Bacillus licheniformis*, and *Bacillus circulans*, survive, germinate, and initiate growth upon cooling [16]. During the cooling and handling phase, the germinated *Bacillus* proliferate rapidly and are joined by environmental lactic acid bacteria (LAB) (*Enterococcus faecium*) and Staphylococci introduced via utensils or ambient air [17]. In the primary solid-state (alkaline) fermentation over 24–72 h, proteolytic *Bacillus* species dominate, hydrolyzing proteins into peptides and ammonia, thereby raising the pH to approximately 8.5, while minor yeasts such as *Saccharomyces cerevisiae* and residual Enterobacteriaceae decline [18]. Finally, during maturation (72–120 h), the microbial community stabilizes around the *Bacillus* consortium, with pathogens such as *Bacillus cereus* or *Staphylococcus aureus* persisting only under conditions of poor hygiene [8]. However, *kantong* undergoes acidic fermentation to produce organic acids, particularly lactic acid. Hence, it is dominated by LAB and yeasts that thrive in acidic medium [9]. For a more defined fermented product with beneficial microbiota, starter cultures must be employed in the fermentation process. This research focused on the use of selective media for microbial isolation and sequencing of the ribosomal RNA gene for the characterization and identification of microorganisms involved in the fermentation of kapok seeds to produce kantong. The study reports the presence of LAB, enterobacteria, and yeasts in the fermentation of kapok seeds into kantong.

## 2. Materials and Methods

### 2.1. Sample Collection

Fermented kapok seeds were obtained from two randomly selected production sites in Nyankpala (Site 1: 9.40224261, −0.9835637; Site 2: 9.40224261, −0.98180703) a farming community in the northern region of Ghana. Samples were taken aseptically from the raw material, different fermentation time points, and from the final product. Temperature at the different fermentation time points was measured at the production sites while pH of the samples was measured at the laboratory [9].

### 2.2. Enumeration of Microorganisms

Tenfold serial dilutions were made for each sample, and 1 mL of the dilutions was cultured in the appropriate selective medium. Aerobic mesophilic bacteria were enumerated on nutrient agar (MERCK, Darmstadt, Germany) and incubated at 30°C for 24 h. LAB were enumerated on de Man, Rogosa, and Sharpe agar (MRS) (MERCK, Darmstadt, Germany) and incubated at 30°C for 72–96 h under anaerobic conditions (Anaerocult A, MERCK kGaA, Darmstadt Germany). Yeasts were enumerated on malt extract agar (MEA) (MERCK, Darmstadt, Germany) supplemented with 50 mg/L chlortetracycline and 100 mg/L chloramphenicol to inhibit bacterial growth and incubated at 25°C for 72 h. Enterobacteria were enumerated on violet red bile glucose agar (VRBGA) (OXOID, Basingstoke, England) and incubated at 37°C for 24 h. Growth on plates with colonies between 30 and 300 was selected for counting from the appropriate dilutions, and the colony-forming units (CFUs) were determined. The experiments were carried out in triplicate, and the microbial load was reported as the mean values of the log_10_ CFU/g of the samples with the calculated standard deviations. The isolates were purified on their respective selective media and stored at 4°C for subsequent downstream experiments.

### 2.3. Genotypic Characterization of Microorganisms

Genomic DNA was extracted from each purified isolate using the InstaGene DNA extraction kit (Bio-Rad Laboratories, Sundbyberg, Sweden), following the manufacturer's protocol. For yeasts and LAB, repetitive element PCR (rep-PCR) was performed using DreamTaq Green PCR Master Mix (Thermo Fisher Scientific), nuclease-free water, template DNA, and the GTGs primer, under the cycling conditions described by [19]. Enterobacterial isolates were subjected to M13-PCR typing with the *Escherichia coli* phage–derived PM13 primer (5⁣′GAGGGTGGCGGCTCT 3⁣′) as outlined by [20]. Fingerprint profiles generated by both rep-PCR and M13-PCR were analyzed in BioNumerics 4.50 (Applied Maths, Sint-Martens-Latem, Belgium) using Dice's coefficient of similarity and clustered via the unweighted pair-group method with arithmetic mean algorithm (UPGMA) [21].

Representative bacterial isolates were further characterized by amplification of their 16S rRNA genes using universal primers 0011f (5⁣′-AGA GTT TGA TYM TGG-3⁣′) and 1510r (5⁣′-ACG GYT ACC TTG TTA-3⁣′), while the D1/D2 region of the 26S rRNA gene in selected yeast isolates was amplified with primers NL-1 (5⁣′-GCATATCAATAAGCGGAGGAAAAG-3⁣′) and NL-4 (5⁣′-GGTCCGTGTTTCAAGACGG-3⁣′) [22]. PCR products were purified and Sanger-sequenced by Macrogen, Korea. Raw sequences were manually edited and aligned in Chromas 2.33 (Technelysium) and CLC Main Workbench 6.6.0 (CLC Bio, Aarhus, Denmark). The resulting 16S rRNA sequences were compared to NCBI GenBank type strain entries using BLAST [23], and provisional identifications were assigned to hits with 100% similarity. These assignments were validated against EzBioCloud's EzTaxon database [24], accepting ≥ 99% identity to a type strain.

For phylogenetic reconstruction, three separate maximum-likelihood trees were generated, one each for the yeast, LAB, and Enterobacteriaceae datasets, using RealPhy [25]. The sequence reads were aligned against the appropriate reference genomes, and phylogeny was inferred. The resulting Newick files were imported into iTOL (Interactive Tree of Life) for branch annotation, rooting, and aesthetic editing to produce publication-quality figures [26].

### 2.4. Nucleotide Accession Numbers

The 16S rRNA and 26S rRNA sequences obtained in the present study were deposited in the National Center for Biotechnology Information (NCBI) GenBank database under the accession numbers OK513529–OK513535 for LAB, OK513524–OK513528 for yeasts, and OK504396–OK504399 for enterobacteria. They correspond to the following GenBank entries: *Pediococcus acidilactici* strain 12aSq-v7 16S ribosomal RNA gene, partial sequence; *Pediococcus acidilactici* strain 14bSq-n9 16S ribosomal RNA gene, partial sequence; *Pediococcus pentosaceus* strain 15aSq-j5 16S ribosomal RNA gene, partial sequence; *Pediococcus pentosaceus* strain 16aSq-i2ii 16S ribosomal RNA gene, partial sequence; *Weissella confusa* strain 27aSq-f6i 16S ribosomal RNA gene, partial sequence; *Weissella confusa* strain 29aSq-j3 16S ribosomal RNA gene, partial sequence; *Lactiplantibacillus plantarum* strain 2aSq-s1 16S ribosomal RNA gene, partial sequence; *Cyberlindnera fabianii* strain Y1sq-a2 large subunit ribosomal RNA gene, partial sequence; *Pichia kudriavzevii* strain Y16sq-l3 large subunit ribosomal RNA gene, partial sequence; *Nakaseomyces glabratus* strain Y17sq-e8 large subunit ribosomal RNA gene, partial sequence; *Saccharomyces cerevisiae* strain Y11sq-i3 large subunit ribosomal RNA gene, partial sequence; *Enterobacter cloacae* strain X14sq 16S ribosomal RNA gene, partial sequence; *Escherichia coli* strain X23sq2 16S ribosomal RNA gene, partial sequence; *Klebsiella pneumoniae* strain X26sq2 16S ribosomal RNA gene, partial sequence; and *Enterococcus faecium* strain X8sq2 16S ribosomal RNA gene, partial sequence (see Supporting Information for complete sequence data).

## 3. Results

### 3.1. Microbial Load, pH, and Temperature of *Kantong* Fermentation

At Production Site 1 (Table 1), the microbial load for aerobic mesophilic bacteria (AMB) ranged from 5.65 ± 0.06 log_10_CFU/g at the start of fermentation to 9.14 ± 0.1 log_10_CFU/g after fermentation. It however dropped to 8.07 ± 0.1 log_10_CFU/g in the final product. A tenfold increase in microbial load was observed every 6 h during the fermentation period up to 30 h, after which microbial growth seemed to enter a stationary phase all through to 48 h. A decrease in microbial load in the dp is an indication of microbial loss because of the drying process. Further processing of the dp by pounding in the household mortar may have reinoculated the product with aerobic mesophiles, leading to a tenfold increase in the microbial load in the final product.

A similar trend was observed with the LAB, with a load of 5.86 ± 0.03 log_10_CFU/g at the start of fermentation and a tenfold increase 6-hourly up to 30 h of fermentation. The drying and further processing resulted in a final load of 7.81 ± 0.06 log_10_CFU/g in the finished product. The yeasts, on the other hand, had a lower a load of 3.89 ± 0.04 log_10_CFU/g at the start of fermentation, compared to the LAB and AMB. Also, a tenfold increase was observed only after 12 h of fermentation, with a further decrease in microbial load at 24 and 30 h. The low yeast load could be attributed to the fermentation temperature of the product, which ranged from 28°C to 38°C. There were no yeasts enumerated from the final product.

Nyankpala Site 2 had a similar pattern in microbial load as Site 1. The overall pH, from both sites, dropped during the fermentation process from about 6.7 to 4.6.

As evident in Tables 1 and 2, *Enterococcus faecium* was observed from the start of fermentation at Site 1 and persisted through to the final product; however, it was not observed at Site 2. This could mean it was an opportunistic pathogen that can be eliminated with the practice of good hygienic processes in the production of kantong. Other nonspore-forming bacteria, including *Acinetobacter baumannii*, *Enterobacter cloacae*, *Escherichia coli*, and *Klebsiella pneumoniae*, were observed at different stages of fermentation from both sites, with percentage occurrences ranging from 7% to 46% of the total number of isolates from a particular plate. However, they were not observed in the final product. *Pediococcus acidilactici* was the predominant LAB from both production sites with 100% occurrence in the final product at Site 2. *Weissella confusa* and *Weissella paramesenteroides* occurred at different stages of production but not in the final product. *Lactiplantibacillus plantarum* was only observed in the cassava flour; however, *Pediococcus pentosaceus* was observed through the fermentation process from both sites and in the final product from Site 1.

Yeasts were found only during the fermentation process but not in the starting material and the final product. *Cyberlindnera fabianii* was observed as the predominant yeast with *Nakaseomyces glabratus* and *Pichia kudriavzevii* occurring at both sites. *Saccharomyces cerevisiae* was only observed at 30 h at Site 1.

### 3.2. Identification of LAB

**Figure 1:**
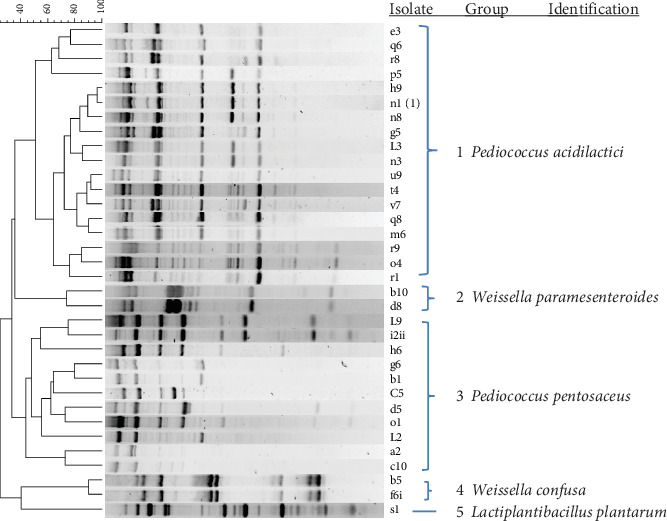
Dendrogram obtained by cluster analysis of rep-PCR (GTG_5_) profiles of representative lactic acid bacteria obtained during *kantong* production at two production sites. The dendrogram is based on Dice's coefficient of similarity with the unweighted pair group method with arithmetic average clustering algorithm. They have been identified by 16S rRNA gene sequencing.

A total of 190 Gram-positive, catalase-negative isolates from MRS medium were presumptively considered LAB. Isolates were genotypically grouped by rep-PCR fingerprinting and clustered into five groups (Figure 1).

The isolates in Group 1 (114 isolates) were identified as *Pediococcus acidilactici* based on their 16S rRNA gene sequence (100% similarity to GenBank sequences and 99.8% similarity in EzTaxon).

Group 2 isolates (5 isolates) were identified as *Weissella paramesenteroides* (100% similarity to GenBank, 99% similarity EzTaxon).

Group 3 isolates (61 isolates) were found to belong to *Pediococcus acidilactici* and *Pediococcus pentosaceus* (100% similarity to GenBank sequences); however, a 99.7% similarity in EzTaxon and a gyrA gene sequence with 99.8% similarity consequently led to the identification of the isolates as *Pediococcus pentosaceus*.

Group 4 isolates (7 isolates) were identified as *Weissella confusa* based on their 16S rRNA gene sequence (100% similarity to GenBank sequences and 100% EzTaxon).

Group 5 (3 isolates) were identified as *Lactiplantibacillus plantarum* based on their 16S rRNA gene sequences (100% similarity to GenBank sequences).

### 3.3. Identification of Enterobacteria

**Figure 2:**
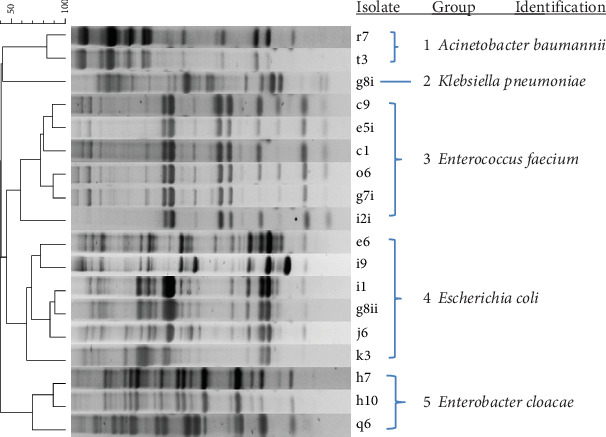
Dendrogram obtained by cluster analysis of M13 PCR profiles of representative enterobacteria isolated during *kantong* production at two production sites. The dendrogram is based on Dice's coefficient of similarity with the unweighted pair group method with arithmetic average clustering algorithm. They have been identified by 16S rRNA gene sequencing.

The nonspore-forming aerobic mesophiles (53 isolates) were grouped using M13-PCR fingerprinting into five (Figure 2). The isolates were identified based on their 16S rRNA sequences as *Acinetobacter baumannii* (Group 1; 8 isolates), *Klebsiella pneumoniae* (Group 2; 5 isolates), *Enterococcus faecium* (Group 3; 16 isolates), *Escherichia coli* (Group 4; 19 isolates), and *Enterobacter cloacae* (Group 5; 5 isolates).

### 3.4. Identification of Yeasts

**Figure 3:**
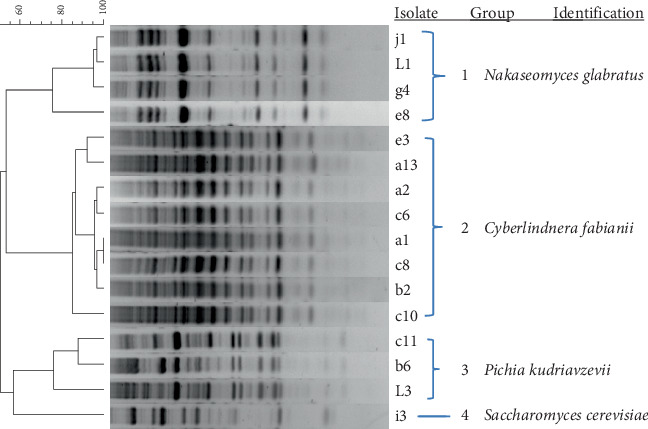
Dendrogram obtained by cluster analysis of rep-PCR (GTG_5_) profiles of representative yeast isolates obtained during *kantong* production at two production sites. The dendrogram is based on Dice's coefficient of similarity with the unweighted pair group method with arithmetic average clustering algorithm. They have been identified by 16S rRNA gene sequencing.

In total, 39 yeast isolates were obtained from all samples. Based on their cluster analysis, isolates were grouped according to their rep-PCR fingerprint pattern as shown in Figure 3. Four species were identified based on the sequencing of the D1/D2 region of the 26S rRNA gene: *Nakaseomyces glabratus* (7 isolates), *Cyberlindnera fabianii* (25 isolates), *Pichia kudriavzevii* (5 isolates), and *Saccharomyces cerevisiae* (2 isolates). All sequences showed high (100%) similarities to sequences in the GenBank.

### 3.5. Phylogenetic Analysis

**Figure 4:**
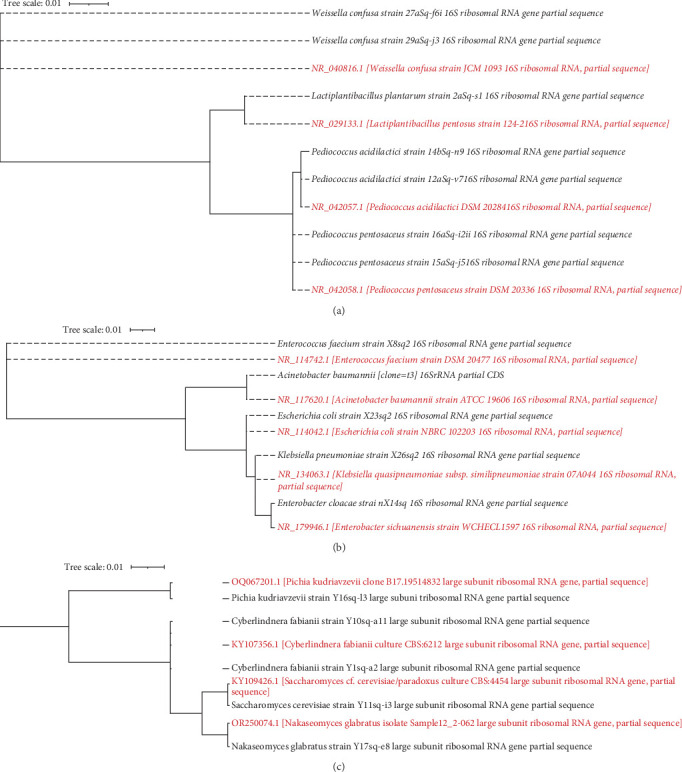
Molecular phylogenetic tree showing the relationship of the sampled strains of lactic acid bacteria, enterobacteria, yeasts, and selected reference strains. The figure presents maximum-likelihood phylogenetic trees comparing our isolates (black labels) to reference type strain sequences (red labels) based on ribosomal RNA gene data. (a) Lactic acid bacteria inferred from nearly full-length 16S rRNA sequences, (b) Enterobacteriaceae from 16S rRNA, and (c) yeast relationships using the D1/D2 domain of the 26S rRNA gene. All three trees were constructed in RealPhy and edited in iTOL; horizontal branch lengths are proportional to genetic distance (scale bar = 0.01 substitutions/site).

In the LAB phylogenetic tree (Figure 4a), *Pediococcus acidilactici* isolates (strains 12aSq-v7 and 14bSq-n9) from this study were seen to cluster tightly with *Pediococcus acidilactici* DSM 20284 type strain, while *Pediococcus pentosaceus* isolates (15aSq-j5 and 16aSq-i2ii) clustered with *Pediococcus pentosaceus* DSM 20336*. Lactiplantibacillus plantarum* strain 2aSq-s1 fell within the larger *Lactiplantibacillus plantarum/pentosus* clade, and two *Weissella confusa* strains (27aSq-f6i and 29aSq-j3) nested firmly alongside *Weissella confusa* JCM 1093. In the Enterobacteriaceae phylogenetic tree (Figure 4b), *Enterococcus faecium* X8sq2 aligned with *Enterococcus faecium* DSM 20477, *Acinetobacter baumannii* clustered with its ATCC 19606 counterpart, *Escherichia coli* X23sq2 grouped with the K-12 lineage, and both *Klebsiella pneumoniae* X26sq2 and *Enterobacter cloacae* X14sq2 formed distinct clades with their respective type strains. The yeast phylogenetic tree (Figure 4c) shows *Pichia kudriavzevii* Y16sq-l3 closely associated with environmental clone B17, *Cyberlindnera fabianii* Y10sq-a11 with the CBS 6212 reference strain, *Nakaseomyces glabratus* Y17sq-e8 clustered with OR250074.1 clinical strain, and *Saccharomyces cerevisiae* Y11sq-i3 in a well-supported clade as seen in Figure 4.

## 4. Discussion

This study provides a comprehensive characterization of selected microbiota and fermentation dynamics of kantong, an acid-fermented seed condiment derived from *Ceiba pentandra* in northern Ghana. During fermentation at both production sites, the pH of the *kantong* matrix decreased significantly from an average of 6.7 to approximately 4.6, thereby confirming that *kantong* undergoes acidic rather than alkaline proteolysis [27]. This behavior contrasts with other West African seed condiments such as *dawadawa* and *ugba*, which are classic alkaline fermentations reaching pH levels of 8.0–8.5, highlighting the unique role of lactic acid production in *kantong*.

In microbial fermentations, both pH and temperature act as critical selective pressures that determine which organisms grow, how quickly they proliferate, and what metabolic end-products accumulate [28]. In acidic fermentations, the drop in pH to between 3.5 and 5.0 favors acid-tolerant LAB such as *Lactobacillus*, *Leuconostoc*, and *Pediococcus*, which can reach densities of 10^8^–10^9^ CFU/g, while inhibiting spoilage and enteric bacteria [29]. Mesophilic temperatures (30°C–37°C) accelerate LAB growth and acid production, whereas thermophilic conditions (> 45°C) impair LAB and may allow heat-resistant spore-formers to persist [30, 31]. Conversely, in alkaline fermentations, proteolysis liberates ammonia and raises pH above 8.0, selecting for alkaliphilic, proteolytic *Bacillus* species (e.g., *B. subtilis*, *B. licheniformis*, and *Bacillus pumilus*) which dominate at counts of 10^7^–10^8^ CFU/g; temperatures around 30°C–40°C optimize their protease activity, while lower or higher extremes slow their growth or enhance sporulation, respectively [32].

In this study, the acidification in *kantong* can be attributed, in part, to the inclusion of fermented cassava flour, which enriches the fermenting food matrix with starch and supports amylolytic LAB. As these LAB degrade starch to fermentable sugars, lactic acid accumulates and drives down pH [33]. Concurrently, the observed 10°C rise in temperature during the first 6–12 h of fermentation likely reflects intensified microbial respiration, creating a self-reinforcing mesophilic environment that favors rapid growth of fermentative organisms [34], and as seen in the study optimal fermentation temperatures between 28°C and 38°C, consistent with the predominance of mesophilic LAB and yeasts were recorded.

Microbial enumeration showed that yeast populations increased at temperatures above 35°C, suggesting that indigenous strains (notably *Cyberlindnera fabianii*, *Pichia kudriavzevii*, and *Nakaseomyces glabratus*) possess remarkable thermotolerance and may hold biotechnological potential for high-temperature fermentations. *Nakaseomyces glabratus* (previously known as *Candida glabrata*) can be found in some African fermented foods, and though it is known to ferment trehalose, it is not generally considered a major player in fermented foods but rather an opportunistic pathogen [35]. *Cyberlindnera fabianii* is known to increase the flavor content of fermented foods and improve the nutritional value of fermented plant-based foods by increasing the amount of phenolic compounds and improving free radical scavenging activity. Technologically, when cocultured with *Saccharomyces cerevisiae*, *Cyberlindnera fabianii* can be used as a starter culture in traditional rice-based fermentations [36], and although *Saccharomyces cerevisiae* was detected only transiently (30 h, Site 1), its known capacity to degrade phytate could enhance mineral bioavailability in the final product [37].

Despite the acidified environment, a diversity of Enterobacteriaceae appeared during early fermentation: *Enterobacter cloacae*, *Acinetobacter baumannii*, *Escherichia coli*, *Klebsiella pneumoniae,* and *Enterococcus faecium*, reflecting the household-level processing and occasional lapses in hygiene [38]. While *Klebsiella pneumoniae* is a human pathogen, it plays a role in certain fermentation processes and can be used in some biotechnological applications, particularly in the production of 1,3-propanediol (PDO) and 2,3-butanediol (BDO) which are used in various industrial applications like bioplastics, solvents, and antifreeze [39]. *Acinetobacter baumannii*, primarily known as a hospital-acquired pathogen, can be involved in flavor development and contribute to the production of enzymes such as lipases and proteases, which break down food substrates [40]. These opportunistic pathogens did not persist in the final product (except for *Enterococcus faecium* at Site 1), likely due to the inhibitory effect of low pH and metabolic by-products on neutralophilic organisms. Nevertheless, their transient presence highlights the need for improved sanitary measures and the adoption of defined starter cultures to ensure product safety.

LAB dominated throughout fermentation, with isolates of *Pediococcus acidilactici* and *Pediococcus pentosaceus* consistently recovered from both process and final samples. *Weissella confusa*, *Weissella paramesenteroides*, and *Lactiplantibacillus plantarum* were detected during early stages but were not found at completion. This is consistent with research that shows the succession of LAB in fermented foods such as kimchi, where *Weissella* and *Leuconostoc* dominate early on during the fermentation, while *Pediococcus* becomes dominant later [41, 42]. This shift is influenced by factors like pH, temperature, and available nutrients, implying that only the most acid-tolerant LAB remain at low pH. Their metabolic activities, particularly lactic and short-chain fatty acid production, likely contribute to pathogen suppression and the characteristic tangy flavor of kantong [43].

The isolated strains contribute synergistically to the sensory attributes of various fermented products and, as such, *kantong*. *Pediococcus acidilactici* and *Pediococcus pentosaceus* produce diacetyl and acetoin, imparting buttery and creamy notes, and exopolysaccharides that enhance mouthfeel [44]. *Lactiplantibacillus plantarum* synthesizes varied organic acids and volatile esters, adding fruity and floral nuances [45]. Thermotolerant yeasts such as *Pichia kudriavzevii* and *Cyberlindnera fabianii* generate higher alcohols and esters via alcohol dehydrogenase and esterase activities, enriching the condiment's aroma profile [46]. Together, this consortium balances acidity, umami, and aromatic complexity, defining the distinctive flavor of kantong.

From the Phylogenetic analysis, maximum-likelihood phylogenies confirm that the core LAB driving *kantong*'s acidification are indeed *Pediococcus acidilactici*, *Pediococcus pentosaceus*, and *Lactiplantibacillus plantarum*: each of the *kantong* isolates clustered with its respective type strain (Figure 4a), supporting the species assignments based on 16S rRNA sequence. Likewise, the Enterobacteriaceae isolates (*Enterobacter cloacae*, *Klebsiella pneumoniae*, *Escherichia coli*, and *Acinetobacter baumannii*) and the facultative anaerobe *Enterococcus faecium* fall into well-supported clades with their reference sequences (Figure 4b), validating their transient occurrence early in fermentation. The yeast phylogeny (Figure 4c) places the *Pichia kudriavzevii* and *Cyberlindnera fabianii* strains within clades of known thermotolerant type strains and confirmed the identity of *Nakaseomyces glabratus* and the occasionally occurring *Saccharomyces cerevisiae* isolate.

Although these phylogenetic trees do not exhibit functionality, they ensure the robustness of the taxonomic identifications in the study. By comparing the isolates from this study to type strains from recognized culture collection, future efforts in starter culture development or functional assays can focus on the appropriate organisms.

This study employed agar plate culturing method for the characterization of microbial strains. While this is useful for isolating and studying specific microbes, it is inadequate for comprehensive microbial community profiling. For future research, modern culture-independent techniques, such as high-throughput sequencing and metagenomics, which offer a complete and more accurate picture of microbial diversity and function can be applied to capture the full breadth of *kantong's* microbial consortia, including unculturable or low-population members that potentially contribute to flavor, safety, or nutritional attributes [47]. Microorganisms isolated from this study give an overview of the culturable strains associated with fermented kapok seeds, *kantong.*

Overall, our findings demonstrate that kantong fermentation represents a mixed-culture acid process, driven primarily by mesophilic LAB and supported by thermotolerant yeasts, with transient colonization by facultative pathogens. To standardize quality and enhance safety, we recommend the development of a starter culture consortium comprising *Pediococcus acidilactici*, *Pediococcus pentosaceus*, and *Lactiplantibacillus plantarum*, together with strict hygienic practices at the household level. Such measures will help ensure the reproducible production of safe, flavorful kantong for local and broader markets.

## 5. Conclusion

The spontaneous fermentation of kapok seeds to produce kantong is an acid fermentation process associated with low pH and uncontrolled temperature during the fermentation process. The microecology of this fermented product reveals the presence of yeasts, LAB, and enterobacteria with relatively high microbial load. Although this makes it a reservoir of microorganisms with potential biotechnological and health-promoting applications, it is also a habitat for pathogenic microorganisms, as seen in the study, and this is characteristic of several investigated fermented foods of African origin. However, precise identification of the strains involved in the production of kantong has been carried out using molecular methods, thus creating room for further investigations leading to the safe production of kantong using the appropriate strains for starter culture development.

## Figures and Tables

**Table 1:** Succession of nonspore-forming bacteria and yeasts during kapok seed fermentation for *kantong* production. pH, temperature, and percentage distributions of the microorganisms were determined (Nyankpala Site 1 9.40224261, −0.9835637).

**Sampling point**	**sd**	**sf**	**cf**	**0 h**	**6** h	**12** h	**24** h	**30 h**	**48** h	**dp**	**fp**
pH	ND	6.74 ± 0.03	6.12 ± 0.02	6.07 ± 0.01	5.22 ± 0.03	5.2 ± 0.17	4.83 ± 0.02	4.75 ± 0.03	4.85 ± 0.02	4.74 ± 0.03	4.65 ± 0.02
Temperature (°C)	ND	ND	ND	28.2 ± 0.0	38.0 ± 0.11	36.5 ± 0.0	27.9 ± 0.1	30.7 ± 0.3	29.2 ± 0.0	ND	ND
AMB (log_10_CFU/g)	5.52 ± 0.03	5.61 ± 0.1	5.6 ± 0.02	5.65 ± 0.06	6.86 ± 0.05	7.62 ± 0.05	8.72 ± 0.1	9.30 ± 0.1	9.14 ± 0.1	7.07 ± 0.1	8.07 ± 0.1
Identity/occurrence (%)^a^											
Enterobacteria											
*Enterobacter cloacae*	—	11	—	—	—	—	—	—	—	—	—
*Acinetobacter baumannii*	—	11	—	—	—	—	—	—	14	14	—
*Enterococcus faecium*	—	—	—	13	50	17	7	7	—	29	11
*Escherichia coli*	—	—	—	—	6	17	40	38	31	—	—
*Klebsiella pneumoniae*	—	—	—	—	—	17	7	—	—	—	—
Lactic acid bacteria (log_10_CFU/g)	3.36 ± 0.05	4.92 ± 0.03	5.84 ± 0.02	5.86 ± 0.03	6.95 ± 0.02	7.42 ± 0.09	8.83 ± 0.03	9.69 ± 0.03	9.71 ± 0.05	8.90 ± 0.15	7.81 ± 0.06
*Pediococcus acidilactici*	100	100	—	—	14	75	88	20	30	88	67
*Weissella paramesenteroides*	—	—	14	—	—	—	—	—	—	12	—
*Pediococcus pentosaceus*	—	—	43	100	86	25	12	80	60	—	33
*Weissella confusa*	—	—	—	—	—	—	—	—	10	—	—
*Lactiplantibacillus plantarum*	—	—	43	—	—	—	—	—	—	—	—
Yeasts (log_10_CFU/g)	—	—	—	3.89 ± 0.04	3.73 ± 0.03	4.52 ± 0.18	4.22 ± 0.13	3.77 ± 0.09	3.59 ± 0.05	—	—
*Pichia kudriavzevii*	—	—	—	8	10	—	—	—	—	—	—
*Cyberlindnera fabianii*	—	—	—	92	72	50	33	—	—	—	—
*Nakaseomyces glabratus*	—	—	—	—	18	50	67	—	—	—	—
*Saccharomyces cerevisiae*	—	—	—	—	—	—	—	100	—	—	*—*

*Note:* 0–48 h: fermentation period.

Abbreviations: cf, cassava flour; dp, dried pellet; fp, final product (*kantong*); sd, seed; sf, seed flour.

^a^Percentage occurrence—calculated as percentage of identified enterobacteria, LAB, or yeast species relative to total number of isolates on a plate from a particular sampling point.

**Table 2:** Succession of nonspore-forming bacteria and yeasts during kapok seed fermentation for *kantong* production. pH, temperature, and percentage distributions of the microorganisms were determined (Nyankpala Site 2 9.40224261, −0.98180703).

**Sampling point**	**sd**	**sf**	**cf**	**0** h	**6** h	**12** h	**24** h	**30** h	**48** h	**dp**	**fp**
pH		6.64 ± 0.03	5.94 ± 0.03	5.97 ± 0.03	5.75 ± 0.02	5.88 ± 0.01	5.11 ± 0.01	5.01 ± 0.02	4.85 ± 0.02	4.73 ± 0.02	4.72 ± 0.01
Temperature (°C)				28.1 ± 0.1	37.5 ± 0.0	35.9 ± 0.1	29.0 ± 0.0	30.1 ± 0.1	29.3 ± 0.2		
AMB (log_10_CFU/g)	3.44 ± 0.1	4.91 ± 0.04	4.42 ± 0.1	4.72 ± 0.04	4.79 ± 0.03	6.87 ± 0.02	7.70 ± 0.1	9.26 ± 0.04	9.08 ± 0.04	7.85 ± 0.02	8.9 ± 0.03
Identity/occurrence (%)^a^											
Enterobacteria											
*Acinetobacter baumannii*	—	14	23	—	—	—	—	—	—	—	—
*Enterobacter cloacae*	—	—	—	—	—	—	46	—	—	—	—
*Enterococcus faecium*	—	—	—	—	—	—	—	—	—	—	—
*Escherichia coli*	—	—	—	—	—	—	—	18	—	—	—
*Klebsiella pneumoniae*	—	—	—	—	—	—	18	9	—	—	—
Lactic acid bacteria (log_10_CFU/g)	3.36 ± 0.05	5.62 ± 0.03	6.43 ± 0.07	6.78 ± 0.04	6.98 ± 0.01	7.41 ± 0.06	8.52 ± 0.03	9.49 ± 0.04	9.55 ± 0.03	8.60 ± 0.03	7.53 ± 0.05
*Pediococcus acidilactici*	100	100	100	—	29	67	11	37	60	80	100
*Pediococcus pentosaceus*	—	—	—	42	43	11	89	50	40	20	—
*Weissella paramesenteroides*	—	—	—	29	14	—	—	—	—	—	—
*Weissella confusa*	—	—	—	29	14	22	—	13	—	—	—
*Lactiplantibacillus plantarum*	—	—	—	—	—	—	—	—	—	—	—
Yeast (log_10_CFU/g)				4.34 ± 0.07	6.49 ± 0.09	6.25 ± 0.06	6.77 ± 0.08	4.28 ± 0.15	4.77 ± 0.11		
*Pichia kudriavzevii*	—	—	—	33	—	50	—	—	50	—	—
*Cyberlindnera fabianii*	—	—	—	67	—	50	—	100	—	—	—
*Nakaseomyces glabratus*	—	—	—	—	—	—	—	—	50	—	—
*Saccharomyces cerevisiae*	—	—	—	—	—	—	—	—	—	—	—

*Note:* 0–48 h: fermentation period.

Abbreviations: cf, cassava flour; dp, dried pellet; fp, final product (*kantong*); sd, seed; sf, seed flour.

^a^Percentage occurrence—calculated as percentage of enterobacteria, LAB, or yeast occurring at a particular sampling point.

## Data Availability

The data that support the findings of this study are available from the corresponding author upon reasonable request.

## Supporting Information

Supporting Information Additional supporting information can be found online in the Supporting Information section. Additional datasets comprising nucleotide sequences of ribosomal RNA genes of microbial strains isolated from kantong and have been deposited in the National Center for Biotechnology Information (NCBI) GenBank database can be found in the supporting information.

## References

[1] Obafemi Y. D., Oranusi S. U., Ajanaku K. O., Akinduti P. A., Leech J., Cotter P. D. (2022). African Fermented Foods: Overview, Emerging Benefits, and Novel Approaches to Microbiome Profiling. *Npj Science of Food*.

[2] Praveen M., Brogi S. (2025). Microbial Fermentation in Food and Beverage Industries: Innovations, Challenges, and Opportunities. *Food*.

[3] Abbaspour N. (2024). Fermentation’s Pivotal Role in Shaping the Future of Plant-Based Foods: An Integrative Review of Fermentation Processes and Their Impact on Sensory and Health Benefits. *Applied Food Research*.

[4] Siddiqui S. A., Erol Z., Rugji J. (2023). An Overview of Fermentation in the Food Industry - Looking Back From a New Perspective. *Bioresources and Bioprocessing*.

[5] Schoustra S., van der Zon C., Groenenboom A. (2022). Microbiological Safety of Traditionally Processed Fermented Foods Based on Raw Milk, the Case of Mabisi From Zambia. *LWT*.

[6] Dimidi E., Cox S. R., Rossi M., Whelan K. (2019). Fermented Foods: Definitions and Characteristics, Impact on the Gut Microbiota and Effects on Gastrointestinal Health and Disease. *Nutrients*.

[7] Leeuwendaal N. K., Stanton C., O’Toole P. W., Beresford T. P. (2022). Fermented Foods, Health and the Gut Microbiome. *Nutrients*.

[8] Olasupo N. A., Okorie P. C., Olasupo N. A., Okorie P. C. (2019). African Fermented Food Condiments: Microbiology Impacts on Their Nutritional Values. *Frontiers and New Trends in the Science of Fermented Food and Beverages*.

[9] Kpikpi E. N., Thorsen L., Glover R., Dzogbefia V. P., Jespersen L. (2014). Identification of Bacillus Species Occurring in Kantong, an Acid Fermented Seed Condiment Produced in Ghana. *International Journal of Food Microbiology*.

[10] Kpikpi E. N., Glover R., Dzogbefia V., Nielsen D. (2010). Isolation of Lactic Acid Bacteria From Kantong, a Condiment Produced From the Fermentation of Kapok (Ceiba pentandra) Seeds and Cassava (Manihot esculentum) Flour. *Report and Opinion*.

[11] Guissou A. W., Parkouda C., Anaïs C. K., Korotimi T., Oboulbiga E. B., Savadogo A. (2020). Fermentation Effect on the Nutrient and Antinutrient Composition of &lt;i&gt;Senegalia macrostachya&lt;/i&gt; and &lt;i&gt;Parkia biglobosa&lt;/i&gt; Seeds: A Comparative Study. *Food and Nutrition Sciences*.

[12] Thorsen L., Kando C. K., Sawadogo H. (2015). Characteristics and Phylogeny of Bacillus cereus Strains Isolated From Maari, a Traditional West African Food Condiment. *International Journal of Food Microbiology*.

[13] Anyogu A., Olukorede A., Anumudu C. (2021). Microorganisms and Food Safety Risks Associated With Indigenous Fermented Foods From Africa. *Food Control*.

[14] Mgbodile F. C., Nwagu T. N. T. (2023). Probiotic Therapy, African Fermented Foods and Food-Derived Bioactive Peptides in the Management of SARS-CoV-2 Cases and Other Viral Infections. *Biotechnology Reports*.

[15] Agunwah I. M., Ogueke C. C., Nwosu J. N., Anyogu A. (2024). Microbiological Evaluation of the Indigenous Fermented Condiment Okpeye Available at Various Retail Markets in the South-Eastern Region of Nigeria. *Heliyon*.

[16] Maojin T., Zheng Z., Ying H., Yanyan H., Liang Z. (2025). Bacterial Spore Inactivation Technology in Solid Foods: A Review. *Journal of Food Protection*.

[17] Wang Y., Wu J., Lv M. (2021). Metabolism Characteristics of Lactic Acid Bacteria and the Expanding Applications in Food Industry. *Frontiers in Bioengineering and Biotechnology*.

[18] Oladeji O. A., Taiwo K. A., Ogidi C. O., Faturoti A. O. (2025). Production, Nutritional Benefits, Limitations and Strategies for Enhancing the National Value of Fermented Native Condiments From Selected Legumes and Wild Seeds in Nigeria. *Discover Food*.

[19] Sato J., Nakayama M., Tomita A., Sonoda T., Hasumi M., Miyamoto T. (2017). Evaluation of Repetitive-PCR and Matrix-Assisted Laser Desorption Ionization-Time of Flight Mass Spectrometry (MALDI-TOF MS) for Rapid Strain Typing of Bacillus coagulans. *PLoS One*.

[20] Ehling-Schulz M., Fricker M., Scherer S. (2004). Bacillus cereus, the Causative Agent of an Emetic Type of Food-Borne Illness. *Molecular Nutrition and Food Research*.

[21] Nielsen D. S., Teniola O. D., Ban-Koffi L., Owusu M., Andersson T. S., Holzapfel W. H. (2007). The Microbiology of Ghanaian Cocoa Fermentations Analysed Using Culture-Dependent and Culture-Independent Methods. *International Journal of Food Microbiology*.

[22] Kubiak J., Morgan A., Kirmaier A., Arnaout R., Riedel S. (2023). Universal PCR for Bacteria, Mycobacteria, and Fungi: A 10-Year Retrospective Review of Clinical Indications and Patient Outcomes. *Journal of Clinical Microbiology*.

[23] Schoch C. L., Ciufo S., Domrachev M. (2020). NCBI Taxonomy: A Comprehensive Update on Curation, Resources and Tools. *Database*.

[24] Yoon S. H., Ha S. M., Kwon S. (2017). Introducing EzBioCloud: A Taxonomically United Database of 16S rRNA Gene Sequences and Whole-Genome Assemblies. *International Journal of Systematic and Evolutionary Microbiology*.

[25] Li Y., Dd W., Ar B. (2014). New Software Tool Automates and Improves the Accuracy of Phylogenetic Inference From Next-Generation Sequencing Data. *Molecular Biology and Evolution*.

[26] Letunic I., Bork P. (2021). Interactive Tree of Life (iTOL) v5: An Online Tool for Phylogenetic Tree Display and Annotation. *Nucleic Acids Research*.

[27] Parkouda C., Nielsen D. S., Azokpota P. (2009). The microbiology of alkaline-fermentation of indigenous seeds used as food condiments in Africa and Asia. *Critical Reviews in Microbiology*.

[28] Sawant S. S., Park H. Y., Sim E. Y., Kim H. S., Choi H. S. (2025). Microbial Fermentation in Food: Impact on Functional Properties and Nutritional Enhancement—A Review of Recent Developments. *Fermentation*.

[29] Vera-Peña M. Y., Rodriguez W. L. R. (2020). Effect of pH on the Growth of Three Lactic Acid Bacteria Strains Isolated From Sour Cream. *Universitas Scientiarum*.

[30] Malleck T., Daufouy G., André S., Broussolle V., Planchon S. (2018). Temperature Impacts the Sporulation Capacities and Spore Resistance of *Moorella thermoacetica*. *Food Microbiology*.

[31] Shi C., Ma J., Wu H. (2022). Evaluation of pH Regulation in Carbohydrate-Type Municipal Waste Anaerobic Co-Fermentation: Roles of pH at Acidic, Neutral and Alkaline Conditions. *Science of the Total Environment*.

[32] Mahakhan P., Apiso P., Srisunthorn K. (2023). Alkaline Protease Production From Bacillus gibsonii6BS15-4 Using Dairy Effluent and Its Characterization as a Laundry Detergent Additive. *Journal of Microbiology and Biotechnology*.

[33] Horlacher N., Oey I., Agyei D. (2023). Learning From Tradition: Health-Promoting Potential of Traditional Lactic Acid Fermentation to Drive Innovation in Fermented Plant-Based Dairy Alternatives. *Fermentation*.

[34] Boantza V. D., Jalobeanu D., Wolfe C. (2020). Fermentation. *Encyclopedia of Early Modern Philosophy and the Sciences*.

[35] Rodríguez-Cerdeira C., Pinto-Almazán R., Saunte D. M. L. (2025). Virulence and Resistance Factors of *Nakaseomyces glabratus* (Formerly Known as *Candida glabrata*) in Europe: A Systematic Review. *Journal of the European Academy of Dermatology and Venereology*.

[36] Liang J., Yuan H., Fei Y. (2024). Effects of Saccharomyces cerevisiae and Cyberlindnera fabianii Inoculation on Rice-Flavor Baijiu Fermentation. *Foods*.

[37] Chuang W. Y., Lin L. J., Hsieh Y. C., Chang S. C., Lee T. T. (2021). Effects of Saccharomyces cerevisiae and Phytase Co-Fermentation of Wheat Bran on Growth, Antioxidation, Immunity and Intestinal Morphology in Broilers. *Animal Bioscience*.

[38] Alves A., Viveiros C., Lopes J. (2021). Microbiological Contamination in Different Food Service Units Associated With Food Handling. *Applied Sciences*.

[39] Mitrea L., Vodnar D. C. (2019). *Klebsiella pneumoniae*–A Useful Pathogenic Strain for Biotechnological Purposes: Diols Biosynthesis Under Controlled and Uncontrolled pH Levels. *Pathogens*.

[40] Muleshkova T., Bazukyan I., Papadimitriou K., Gotcheva V., Angelov A., Dimov S. G. (2025). Exploring the Multifaceted Genus *Acinetobacte*r the Facts, the Concerns and the Opportunities the Dualistic Geuns *Acinetobacter*. *Journal of Microbiology and Biotechnology*.

[41] Lee S. H., Jung J. Y., Jeon C. O. (2015). Source Tracking and Succession of Kimchi Lactic Acid Bacteria During Fermentation. *Journal of Food Science*.

[42] Nugroho D., Thinthasit A., Surya E. (2024). Immunoenhancing and Antioxidant Potentials of Kimchi, an Ethnic Food From Korea, as a Probiotic and Postbiotic Food. *Journal of Ethnic Foods*.

[43] Ayivi R. D., Gyawali R., Krastanov A. (2020). Lactic Acid Bacteria: Food Safety and Human Health Applications. *Dairy*.

[44] Jiang G., He J., Gan L. (2022). Exopolysaccharide Produced by Pediococcus pentosaceus E8: Structure, Bio-Activities, and Its Potential Application. *Frontiers in Microbiology*.

[45] Guan X., Zhao D., Yu T. (2024). Phytochemical and Flavor Characteristics of Mulberry Juice Fermented With Lactiplantibacillus plantarum BXM2. *Food*.

[46] Chamnipa N., Thanonkeo S., Klanrit P., Thanonkeo P. (2018). The Potential of the Newly Isolated Thermotolerant Yeast Pichia kudriavzevii RZ8-1 for High-Temperature Ethanol Production. *Brazilian Journal of Microbiology*.

[47] Gutiérrez-Sarmiento W., Peña-Ocaña B. A., Lam-Gutiérrez A., Guzmán-Albores J. M., Jasso-Chávez R., Ruíz-Valdiviezo V. M. (2022). Microbial Community Structure, Physicochemical Characteristics and Predictive Functionalities of the Mexican Tepache Fermented Beverage. *Microbiological Research*.

