# Supplementary material for: Molecular Characterization of Culturable Yeasts and Nonspore-Forming Bacteria Associated With Fermented Kapok Seeds (Kantong), a Traditional Food Condiment in Ghana
Source: Int J Food Sci. 2025 Jun 23;2025:6452183. doi: 10.1155/ijfo/6452183 (PMC12208756; doi:10.1155/ijfo/6452183)
Supplement: Supporting Information — Additional supporting information can be found online in the Supporting Information section. Additional datasets comprising nucleotide sequences of ribosomal RNA genes of microbial strains isolated from kantong and have been deposited in the National Center for Biotechnology Information (NCBI) GenBank database can be found in the supporting information. [file 6452183.f1.docx]

**Supplementary I**

**Molecular characterization of culturable yeasts and non-spore forming bacteria associated with fermented kapok seeds (*kantong*), a traditional food condiment in Ghana.**

*Elmer Nayra Ametefe*^1^, Line Thorsen^2^, Harry Danwonno^1^, Righteous Kwaku Agoha, Richard L.K. Glover^3^, Victoria Pearl Dzogbefia ^4^, Lene Jespersen ^5^*

Nucleotide sequences of ribosomal RNA genes of microbial strains isolated from kantong.

1. ACCESSION NUMBER OK513529 – OK513535 Lactic Acid Bacteria strains.

OK513529 <https://www.ncbi.nlm.nih.gov/nuccore/OK513529>

**Pediococcus acidilactici strain 12aSq-v7 16S ribosomal RNA gene, partial sequence**

1 tgcagtcgaa cgaacttccg ttaattgatt atgacgtgct tgcactgaat gagattttaa

61 cacgaagtga gtggcggacg ggtgagtaac acgtgggtaa cctgcccaga agcaggggat

121 aacacctgga aacagatgct aataccgtat aacagagaaa accgcctggt tttcttttaa

181 aagatggctc tgctatcact tctggatgga cccgcggcgc attagctagt tggtgaggta

241 acggctcacc aaggcgatga tgcgtagccg acctgagagg gtaatcggcc acattgggac

301 tgagacacgg cccagactcc tacgggaggc agcagtaggg aatcttccac aatggacgca

361 agtctgatgg agcaacgccg cgtgagtgaa gaagggtttc ggctcgtaaa gctctgttgt

421 taaagaagaa cgtgggtgag agtaactgtt cacccagtga cggtatttaa ccagaaagcc

481 acggctaact acgtgccagc agccgcggta atacgtaggt ggcaagcgtt atccggattt

541 attgggcgta aagcgagcgc aggcggtctt ttaagtctaa tgtgaaagcc ttcggctcaa

601 ccgaagaagt gcattggaaa ctgggagact tgagtgcaga agaggacagt ggaactccat

661 gtgtagcggt gaaatgcgta gatatatgga agaacaccag tggcgaaggc ggctgtctgg

721 tctgtaactg acgctgaggc tcgaaagcat gggtagcgaa caggattaga taccctggta

781 gtccatgccg taaacgatga ttactaagtg ttggagggtt tccgcccttc agtgctgcag

841 ctaacgcatt aagtaatccg cctggggagt acgaccgcaa ggttgaaact caaaagaatt

901 gacgggggcc cgcacaagcg gtggagcatg tggtttaatt cgaagctacg cgaagaacct

961 taccaggtct tgacatcttc tgccaaccta agagattagg cgttcccttc ggggacagaa

1021 tgacaggtgg tgcatggttg tcgtcagctc gtgtcgtgag atgttgggtt aagtcccgca

1081 acgagcgcaa cccttattac tagttgccag cattcagttg ggcactctag tgagactgcc

1141 ggtgacaaac cggaggaagg tggggacgac gtcaaatcat catgcccctt atgacctggg

1201 ctacacacgt gctacaatgg atggtacaac gagttgcgaa accgcgaggt ttagctaatc

1261 tcttaaaacc attctcagtt cggactgtag gctgcaactc gcctacacga agtcggaatc

1321 gctagtaatc gcggatcagc atgccgcggt gaatacgttc ccgggccttg tacacaccgc

1381 ccgtcacacc atgagagttt gtaacaccca aagccggtgg ggtaaccttt taggagctag

1441 ccgtctaag

OK513530 <https://www.ncbi.nlm.nih.gov/nuccore/OK513530>

**Pediococcus acidilactici strain 14bSq-n9 16S ribosomal RNA gene, partial sequence**

1 cagtcgaacg aacttccgtt aattgatcat gacgtgcttg cactgaatga gattttaaca

61 cgaagtgagt ggcggacggg tgagtaacac gtgggtaacc tgcccagaag caggggataa

121 cacctggaaa cagatgctaa taccgtataa cagagaaaac cgcctggttt tcttttaaaa

181 gatggctctg ctatcacttc tggatggacc cgcggcgcat tagctagttg gtgaggtaac

241 ggctcaccaa ggcgatgatg cgtagccgac ctgagagggt aatcggccac attgggactg

301 agacacggcc cagactccta cgggaggcag cagtagggaa tctttcccca atggacgcaa

361 gtctgatgga gcaaccgccg cgtgagtgaa gaagggtttc ggctcgtaaa gctctgttgt

421 taaagaagaa cgtgggtgag agtaactgtt cacccagtga cggtatttaa ccagaaagcc

481 acggctaact acgtgccagc agccgcggta atacgtaggt ggcaagcgtt atccggattt

541 attgggcgta aagcgagcgc aggcggtctt ttaagtctaa tgtgaaagcc ttcggctcaa

601 ccgaagaagt gcattggaaa ctgggagact tgagtgcaga agaggacagt ggaactccat

661 gtgtagcggt gaaatgcgta gatatatgga agaacaccag tggcgaaggc ggctgtctgg

721 tctgtaactg acgctgaggc tcgaaagcat gggtagcgaa caggattaga taccctggta

781 gtccatgccg taaacgatga ttactaagtg ttggagggtt tccgcccttc agtgctgcag

841 ctaacgcatt aagtaatccg cctggggagt acgaccgcaa ggttgaaact caaaagaatt

901 gacgggggcc cgcacaagcg gtggagcatg tggtttaatt cgaagctacg ccgaagaacc

961 ttaccaggtc ttgacatctt ctgccaacct aagagattag gcgttccctt cggggacaga

1021 atgacaggtg gtgcatggtt gtcgtcagct cgtgtcgtga gatgttgggt taagtcccgc

1081 aacgagcgca acccttatta ctagttgcca gcattcagtt gggcactcta gtgagactgc

1141 cggtgacaaa ccggaggaag gtggggacga cgtcaaatca tcatgcccct tatgacctgg

1201 gctacacacg tgctacaatg gatggtacaa cgagttgcga aaccgcgagg tttagctaat

1261 ctcttaaaac cattctcagt tcggactgta ggctgcaact cgcctacacg aagtcggaat

1321 cgctagtaat cgcggatcag catgccgcgg tgaatacgtt cccgggcctt gtacacaccg

1381 cccgtcacac catgagagtt tgtaacaccc aaagccggtg gggtaacctt taggagct

//

OK513531 <https://www.ncbi.nlm.nih.gov/nuccore/OK513531>

**Pediococcus pentosaceus strain 15aSq-j5 16S ribosomal RNA gene, partial sequence**

1 tgcagtcgaa cgaacttccg ttaattgatt atgacgtact tgtactgatt gagattttaa

61 cacgaagtga gtggcgaacg ggtgagtaac acgtgggtaa cctgcccaga agtaggggat

121 aacacctgga aacagatgct aataccgtat aacagagaaa accgcatggt tttcttttaa

181 aagatggctc tgctatcact tctggatgga cccgcggcgt attagctagt tggtgaggta

241 aaggctcacc aaggcagtga tacgtagccg acctgagagg gtaatcggcc acattgggac

301 tgagacacgg cccagactcc tacgggaggc agcagtaggg aatcttccac aatggacgca

361 agtctgatgg agcaacgccg cgtgagtgaa gaagggtttc ggctcgtaaa gctctgttgt

421 taaagaagaa cgtgggtaag agtaactgtt tacccagtga cggtatttaa ccagaaagcc

481 acggctaact acgtgccagc agccgcggta atacgtaggt ggcaagcgtt atccggattt

541 attgggcgta aagcgagcgc aggcggtctt ttaagtctaa tgtgaaagcc ttcggctcaa

601 ccgaagaagt gcattggaaa ctgggagact tgagtgcaga agaggacagt ggaactccat

661 gtgtagcggt gaaatgcgta gatatatgga agaacaccag tggcgaaggc ggctgtctgg

721 tctgcaactg acgctgaggc tcgaaagcat gggtagcgaa caggattaga taccctggta

781 gtccatgccg taaacgatga ttactaagtg ttggagggtt tccgcccttc agtgctgcag

841 ctaacgcatt aagtaatccg cctgggggag tacgaccgca aggttgaaac tcaaaagaat

901 tgacgggggc ccgcacaagc ggtggagcat gtggtttaat tcgaagctac gcgaagaacc

961 ttaccaggtc ttgacatctt ctgacagtct aagaagatta gaggttccct tcggggacag

1021 aatgacaggt gggtgcatgg ttgtcgtcag ctcgtgtcgt gagatggttg gggttaagtc

1081 ccgcaacgag cgcaaccctt attactagtt gccagcatta agttgggcac tctagtgaga

1141 ctgccggtga caaaccggag gaaggtgggg acgacgtcaa atcatcatgc cccttatgac

1201 ctgggctaca cacgtgctac aatggatggt acaacgagtc gcgagaccgc gaggttaagc

1261 taatctctta aaaccattct cagttcggac tgtaggctgc aactcgccta cacgaagtcg

1321 gaatcgctag taatcgcgga tcagcatgcc gcggtgaata cgttcccggg ccttgtacac

1381 accgcccgtc acaccatgag agtttgtaac acccaaagcc ggtggggtaa ccttttagga

1441 gctagccgtc taa

//

OK513532 <https://www.ncbi.nlm.nih.gov/nuccore/OK513532>

**Pediococcus pentosaceus strain 16aSq-i2ii 16S ribosomal RNA gene, partial sequence**

1 tgcagtcgaa cgaacttccg ttaattgatt atgacgtact tgtactgatt gagattttaa

61 cacgaagtga gtggcgaacg ggtgagtaac acgtgggtaa cctgcccaga agtaggggat

121 aacacctgga aacagatgct aataccgtat aacagagaaa accgcatggt tttcttttaa

181 aagatggctc tgctatcact tctggatgga cccgcggcgt attagctagt tggtgaggta

241 aaggctcacc aaggcagtga tacgtagccg acctgagagg gtaatcggcc acattgggac

301 tgagacacgg cccagactcc tacgggaggc agcagtaggg aatcttccac aatggacgca

361 agtctgatgg agcaacgccg cgtgagtgaa gaagggtttc ggctcgtaaa gctctgttgt

421 taaagaagaa cgtgggtaag agtaactgtt tacccagtga cggtatttaa ccagaaagcc

481 acggctaact acgtgccagc agccgcggta atacgtaggt ggcaagcgtt atccggattt

541 attgggcgta aagcgagcgc aggcggtctt ttaagtctaa tgtgaaagcc ttcggctcaa

601 ccgaagaagt gcattggaaa ctgggagact tgagtgcaga agaggacagt ggaactccat

661 gtgtagcggt gaaatgcgta gatatatgga agaacaccag tggcgaaggc ggctgtctgg

721 tctgcaactg acgctgaggc tcgaaagcat gggtagcgaa caggattaga taccctggta

781 gtccatgccg taaacgatga ttactaagtg ttgggaaggg tttccgccct tcagtgctgc

841 agctaacgca ttaagtaatc cgcctggggg agtacgaccg caaggttgaa actcaaaaga

901 attgacgggg gcccgcacaa gcggtggagc catggtggtt taattcgaag ctacgcgaag

961 aaccttacca ggtcttgaca tcttctgaca gtctaagaag attagaggtt cccttcgggg

1021 acagaatgac aggtgggtgc atggttgtcg tcagctcgtg tcgtgagaat gttgggttaa

1081 gtcccgcaac gagcgcaacc cttattacta gttgccagca ttaagttggg cactctagtg

1141 agactgccgg tgacaaaccg gaggaaggtg gggacgacgt caaatcatca tgccccttat

1201 gacctgggct acacacgtgc tacaatggat ggtacaacga gtcgcgagac cgcgaggtta

1261 agctaatctc ttaaaaccat tctcagttcg gactgtaggc tgcaactcgc ctacacgaag

1321 tcggaatcgc tagtaatcgc ggatcagcat gccgcggtga atacgttccc gggccttgta

1381 cacaccgccc gtcacaccat gagagtttgt aacacccaaa gccggtgggg taacctttta

1441 ggagctagcc gtc

//

OK313533 <https://www.ncbi.nlm.nih.gov/nuccore/OK513533>

**Weissella confusa strain 27aSq-f6i 16S ribosomal RNA gene, partial sequence**

1 tgcagtcgaa cgctttgtgg ttcactgatt tgaagagctt gctcagatat gacgatggac

61 attgcaaaga gtggcgaacg ggtgagtaac acgtgggaaa cctacctctt agcaggggat

121 aacatttgga aacagatgct aataccgtat aacaatgaca accgcatggt tgttatttaa

181 aagatggttc tgctatcact aagagatggt cccgcggtgc attagctagt tggtaaggta

241 atggcttacc aaggcgatga tgcatagccg agttgagaga ctgatcggcc acaatgggac

301 tgagacacgg cccatactcc tacgggaggc agcagtaggg aatcttccac aatgggcgaa

361 agcctgatgg agcaacgccg cgtgtgtgat gaagggtttc ggctcgtaaa acactgttgt

421 aagagaagaa tgacattgag agtaactgtt caatgtgtga cggtatctta ccagaaagga

481 acggctaaat acgtgccagc agccgcggta atacgtatgt tccaagcgtt atccggattt

541 attgggcgta aagcgagcgc agacggttat ttaagtctga agtgaaagcc ctcagctcaa

601 ctgaggaatt gctttggaaa ctggatgact tgagtgcagt agaggaaagt ggaactccat

661 gtgtagcggt gaaatgcgta gatatatgga agaacaccag tggcgaaggc ggctttctgg

721 actgtaactg acgttgaggc tcgaaagtgt gggtagcaaa caggattaga taccctggta

781 gtccacaccg taaacgatga gtgctaggtg tttgagggtt tccgccctta agtgccgcag

841 ctaacgcatt aagcactccg cctgggggag tacgaccgca aggttgaaac tcaaaggaat

901 tgacggggac ccgcacaagc ggtggagcca tgtggtttaa ttcgaagcaa cgcgaagaac

961 cttaccaggt cttgacatcc cttgacaact ccagaagatg gagcgttccc ttcggggaca

1021 aggtgacagg tggtgcatgg ttgtcgtcag ctcgtgtcgt gagatgttgg gttaagtccc

1081 gcaacgagcg caacccttat tactagttgc cagcattcag ttgggcactc tagtgagact

1141 gccggtgaca aaccggagga aggtggggat gacgtcaaat catcatgccc cttatgacct

1201 gggctacaca cgtgctacaa tggcgtatac aacgagttgc caacccgcga gggtgagcta

1261 atctcttaaa gtacgtctca gttcggattg taggctgcaa ctcgcctaca tgaagtcgga

1321 atcgctagta atcgcggatc agcacgccgc ggtgaatacg ttcccgggtc ttgtacacac

1381 cgcccgtcac accatgagag tttgtaacac ccaaagccgg tggggtaacc ttcgggagcc

1441 agccgtctaa g

//

OK513534 <https://www.ncbi.nlm.nih.gov/nuccore/OK513534>

**Weissella confusa strain 29aSq-j3 16S ribosomal RNA gene, partial sequence**

1 tggcagtcga acgctttgtg gttcaactga tttgaagagc ttgctcagat atgacgatgg

61 acattgcaaa gagtggcgaa cgggtgagta acacgtggga aacctacctc ttagcagggg

121 ataacatttg gaaacagatg ctaataccgt ataacaatga caaccgcatg gttgttattt

181 aaaagatggt tctgctatca ctaagagatg gtcccgcggt gcattagcta gttggtaagg

241 taatggctta ccaaggcgat gatgcatagc cgagttgaga gactgatcgg ccacaatggg

301 actgagacac ggcccatact cctacgggag gcagcagtag ggaatcttcc acaatgggcg

361 aaagcctgat ggagcaacgc cgcgtgtgtg atgaagggtt tcggctcgta aaacactgtt

421 gtaagagaag aatgacattg agagtaactg ttcaatgtgt gacggtatct taccagaaag

481 gaacggctaa atacgtgcca gcagccgcgg taatacgtat gttccaagcg ttatccggat

541 ttattgggcg taaagcgagc gcagacggtt atttaagtct gaagtgaaag ccctcagctc

601 aactgaggaa ttgctttgga aactggatga cttgagtgca gtagaggaaa gtggaactcc

661 atgtgtagcg gtgaaatgcg tagatatatg gaagaacacc agtggcgaag gcggctttct

721 ggactgtaac tgacgttgag gctcgaaagt gtgggtagca aacaggatta gataccctgg

781 tagtccacac cgtaaacgat gagtgctagg tgttttgagg gtttccgccc ttaagtgccg

841 cagctaacgc attaagcact ccgcctgggg gagtacgacc gcaaggttga aactcaaagg

901 aattgacggg gacccgcaca agcggtggaa gcatgtggtt taattcgaag caacgcgaag

961 aaccttacca ggtcttgaca tcccttgaca actccagaag atggagcgtt cccttcgggg

1021 acaaggtgac aggtggttgc atggttgtcg tcagctcgtg tcgtgagaat gttgggttaa

1081 gtcccgcaac gagcgcaacc cttattacta gttgccagca ttcagttggg cactctagtg

1141 agactgccgg tgacaaaccg gaggaaggtg gggatgacgt caaatcatca tgccccttat

1201 gacctgggct acacacgtgc tacaatggcg tatacaacga gttgccaacc cgcgagggtg

1261 agctaatctc ttaaagtacg tctcagttcg gattgtaggc tgcaactcgc ctacatgaag

1321 tcggaatcgc tagtaatcgc ggatcagcac gccgcggtga atacgttccc gggtcttgta

1381 cacaccgccc gtcacaccat gagagtttgt aacacccaaa gccggtgggg taaccttcgg

1441 gagccagccg tctaag

OK513535 <https://www.ncbi.nlm.nih.gov/nuccore/OK513535>

**Lactiplantibacillus plantarum strain 2aSq-s1 16S ribosomal RNA gene, partial sequence**

1 tgcagtcgaa cgaactctgg tattgattgg tgcttgcatc atgatttaca tttgagtgag

61 tggcgaactg gtgagtaaca cgtgggaaac ctgcccagaa gcgggggata acacctggaa

121 acagatgcta ataccgcata acaacttgga ccgcatggtc cgagtttgaa agatggcttc

181 ggctatcact tttggatggt cccgcggcgt attagctaga tggtgaggta acggctcacc

241 atggcaatga tacgtagccg acctgagagg gtaatcggcc acattgggac tgagacacgg

301 cccaaactcc tacgggaggc agcagtaggg aatcttccac aatggacgaa agtctgatgg

361 agcaacgccg cgtgagtgaa gaagggtttc ggctcgtaaa actctgttgt taaagaagaa

421 catatctgag agtaactgtt caggtattga cggtatttaa ccagaaagcc acggctaact

481 acgtgccagc agccgcggta atacgtaggt ggcaagcgtt gtccggattt attgggcgta

541 aagcgagcgc aggcggtttt ttaagtctga tgtgaaagcc ttcggctcaa ccgaagaagt

601 gcatcggaaa ctgggaaact tgagtgcaga agaggacagt ggaactccat gtgtagcggt

661 gaaatgcgta gatatatgga agaacaccag tggcgaaggc ggctgtctgg tctgtaactg

721 acgctgaggc tcgaaagtat gggtagcaaa caggattaga taccctggta gtccataccg

781 taaacgatga atgctaagtg tttggaaggg tttccggccc ttcagtgctg cagctaacgc

841 attaagcatt ccgcctgggg gagtacggcc gcaaggctga aactcaaagg aattgacggg

901 ggcccgcaca agcggtggag ccatggtggt ttaattcgaa gctacgcgaa gaaccttacc

961 aggtcttgac atactatgca aatctaagaa gattagacgt tcccttcggg gacatgggat

1021 acaggtggtg catggttgtc gtcagctcgt gtcgtgagat gttgggttaa gtcccgcaac

1081 gagcgcaacc cttattatca gttgccagca ttaagttggg cactctggtg agactgccgg

1141 tgacaaaccg gaggaaggtg gggatgacgt caaatcatca tgccccttat gacctgggct

1201 acacacgtgc tacaatggat ggtacaacga gttgcgaact cgcgagagta agctaatctc

1261 ttaaagccat tctcagttcg gattgtaggc tgcaactcgc ctacatgaag tcggaatcgc

1321 tagtaatcgc ggatcagcat gccgcggtga atacgttccc gggccttgta cacaccgccc

1381 gtcacaccat gagagtttgt aacacccaaa gtcggtgggg taacctttta ggaaccagcc

1441 g

//

1. ACCESSION NUMBERS OK513524 – OK513528 Yeast strains

OK513524 <https://www.ncbi.nlm.nih.gov/nuccore/OK513524>

**Cyberlindnera fabianii strain Y10sq-a11 large subunit ribosomal RNA gene, partial sequence**

1 ggaggaaaag aaaccaacag ggattgcctc agtaacggcg agtgaagcgg caaaagctca

61 aatttgaaat ctagtacctt cggtgctcga gttgtaattt gaaggtagtt ttctggtgct

121 ggcccttgtc tatgttcctt ggaacaggac gtcatagagg gtgagaatcc cgtctgatgg

181 ggtgtccagt gctttgtaga tttctaccga agagtcgagt tgtttgggaa tgcagctcta

241 agtgggtggt aaattccatc taaagctaaa tactggcgag agaccgatag cgaacaagta

301 cagtgatgga aagatgaaaa gaactttgaa aagagagtga aaaagtacgt gaaattgttg

361 aaagggaagg gtattggatc agacttggtg tcttgtgatt atcttccctt cttgggttgt

421 gcactcgcat ttcactgggc cagcatcggt tcggatggca agataatggc ttgggaatgt

481 ggcactcttc ggagtgtgtt atagcccttg ttgatgttgc ctatctggac cgaggactgc

541 ggcttttgcc taggatgctg gcgtaatgat tcaataccgc ccgtcttgac caacggaacc

OK513525 <https://www.ncbi.nlm.nih.gov/nuccore/OK513525>

**Cyberlindnera fabianii strain Y1sq-a2 large subunit ribosomal RNA gene, partial sequence**

1 ggggaaaaga aaccaacagg gattgcctca gtaacggcga gtgaagcggc aaaagctcaa

61 atttgaaatc tagtaccttc ggtgctcgag ttgtaatttg aaggtagttt tctggtgctg

121 gcccttgtct atgttccttg gaacaggacg tcatagaggg tgagaatccc gtctgatggg

181 gtgtccagtg ctttgtagat ttctaccgaa gagtcgagtt gtttgggaat gcagctctaa

241 gtgggtggta aattccatct aaagctaaat actggcgaga gaccgatagc gaacaagtac

301 agtgatggaa agatgaaaag aactttgaaa agagagtgaa aaagtacgtg aaattgttga

361 aagggaaggg tattggatca gacttggtgt cttgtgatta tcttcccttc ttgggttgtg

421 cactcgcatt tcactgggcc agcatcggtt cggatggcaa gataatggct tgggaatgtg

481 gcactcttcg gagtgtgtta tagcccttgt tgatgttgcc tatctggacc gaggactgcg

541 gcttttgcct aggatgctgg cgtaatgatt caataccgcc cgtct

OK513526 <https://www.ncbi.nlm.nih.gov/nuccore/OK513526>

**Pichia kudriavzevii strain Y16sq-l3 large subunit ribosomal RNA gene, partial sequence**

1 ggaggaaaag aaaccaacag ggattgcctc agtagcggcg agtgaagcgg caagagctca

61 gatttgaaat cgtgctttgc ggcacgagtt gtagattgca ggttggagtc tgtgtggaag

121 gcggtgtcca agtcccttgg aacagggcgc ccaggagggt gagagccccg tgggatgccg

181 gcggaagcag tgaggccctt ctgacgagtc gagttgtttg ggaatgcagc tccaagcggg

241 tggtaaattc catctaaggc taaatactgg cgagagaccg atagcgaaca agtactgtga

301 aggaaagatg aaaagcactt tgaaaagaga gtgaaacagc acgtgaaatt gttgaaaggg

361 aagggtattg cgcccgacat ggggattgcg caccgctgcc tctcgtgggc ggcgctctgg

421 gctttccctg ggccagcatc ggttcttgct gcaggagaag gggttctgga acgtggctct

481 tcggagtgtt atagccaggg ccagatgctg cgtgcgggga ccgaggactg cggccgtgta

541 ggtcacggat gctggcagaa cggcgcaaca ccgcccgtct gaa

OK513527 <https://www.ncbi.nlm.nih.gov/nuccore/OK513527>

**Nakaseomyces glabratus strain Y17sq-e8 large subunit ribosomal RNA gene, partial sequence**

1 gaggaaaaga aaccaactgg gattgcctta gtaacggcga gtgaagcggc aaaagctcaa

61 atttgaaatc tggtaccttt ggtgcccgag ttgtaatttg gagagtacca ctttgggact

121 gtactttgcc tatgttcctt ggaacaggac gtcatggagg gtgagaatcc cgtgtggcga

181 gggtgtcagt tctttgtaaa gggtgctcga agagtcgagt tgtttgggaa tgcagctcta

241 agtgggtggt aaattccatc taaagctaaa tacaggcgag agaccgatag cgaacaagta

301 cagtgatgga aagatgaaaa gaactttgaa aagagagtga aaaagtacgt gaaattgttg

361 aaagggaagg gcatttgatc agacatggtg ttttgcgccc cttgcctctc gtgggcttgg

421 gactctcgca gctcactggg ccagcatcgg ttttggcggc cggaaaaaac ctagggaatg

481 tggctctgcg cctcggtgta gagtgttata gccctgggga atacggccag ccgggaccga

541 ggactgcgat acttgttatc taggatgctg gcataatggt tatatgccgc ccg

OK513528 <https://www.ncbi.nlm.nih.gov/nuccore/OK513528>

**Saccharomyces cerevisiae strain Y11sq-i3 large subunit ribosomal RNA gene, partial sequence**

1 gaggaaaaga aaccaaccgg gattgcctta gtaacggcga gtgaagcggc aaaagctcaa

61 atttgaaatc tggtaccttc ggtgcccgag ttgtaatttg gagagggcaa ctttggggcc

121 gttccttgtc tatgttcctt ggaacaggac gtcatagagg gtgagaatcc cgtgtggcga

181 ggagtgcggt tctttgtaaa gtgccttcga agagtcgagt tgtttgggaa tgcagctcta

241 agtgggtggt aaattccatc taaagctaaa tattggcgag agaccgatag cgaacaagta

301 cagtgatgga aagatgaaaa gaactttgaa aagagagtga aaaagtacgt gaaattgttg

361 aaagggaagg gcatttgatc agacatggtg ttttgtgccc tctgctcctt gtgggtgggg

421 gaatctcgca tttcactggg ccagcatcag ttttggtggc aggataaatg cataggaatg

481 tagcttgcct cggtaagtgt tatagcctgt gggaatactg ccagctggga ctgaggactg

541 cgacgtaagt caaggatgct ggcataatgg ttatatgccg cccgtct

1. ACCESSION NUMBERS OK504396 – OK504399 Enterobacteria strains

OK504396 <https://www.ncbi.nlm.nih.gov/nuccore/OK504396>

**Enterobacter cloacae strain X14sq 16S ribosomal RNA gene, partial sequence**

1 tgcagtcgaa cggtagcaca gagagcttgc tctcgggtga cgagtggcgg acgggtgagt

61 aatgtctggg aaactgcctg atggaggggg ataactactg gaaacggtag ctaataccgc

121 ataacgtcgc aagaccaaag agggggacct tcgggcctct tgccatcaga tgtgcccaga

181 tgggattagc tagtaggtgg ggtaacggct cacctaggcg acgatcccta gctggtctga

241 gaggatgacc agccacactg gaactgagac acggtccaga ctcctacggg aggcagcagt

301 ggggaatatt gcacaatggg cgcaagcctg atgcagccat gccgcgtgta tgaagaaggc

361 cttcgggttg taaagtactt tcagcgggga ggaaggtgtt gaggttaata acctcagcaa

421 ttgacgttac ccgcagaaga agcaccggct aactccgtgc cagcagccgc ggtaatacgg

481 agggtgcaag cgttaatcgg aattactggg cgtaaagcgc acgcaggcgg tctgtcaagt

541 cggatgtgaa atccccgggc tcaacctggg aactgcattc gaaactggca ggctagagtc

601 ttgtagaggg gggtagaatt ccaggtgtag cggtgaaatg cgtagagatc tggaggaata

661 ccggtggcga aggcggcccc ctggacaaag actgacgctc aggtgcgaaa gcgtggggag

721 caaacaggat tagataccct ggtagtccac gccgtaaacg atgtcgactt ggaggttgtg

781 cccttgaggc gtggcttccg gagctaacgc gttaagtcga ccgcctgggg agtacggccg

841 caaggttaaa actcaaatga attgacgggg gcccgcacaa gcggtggagc atgtggttta

901 attcgatgca acgcgaagaa ccttacctac tcttgacatc cagagaactt tccagagatg

961 gattggtgcc ttcgggaact ctgagacagg tgctgcatgg ctgtcgtcag ctcgtgttgt

1021 gaaatgttgg gttaagtccc gcaacgagcg caacccttat cctttgttgc cagcggttag

1081 gccgggaact caaaggagac tgccagtgat aaactggagg aaaaggtggg gatgacgtca

1141 agtcatcatg gcccttacga gtagggctac acacgtgcta caatggcgca tacaaagaga

1201 agcgacctcg cgagagcaag cggacctcat aaagtgcgtc gtagtccgga ttggagtctg

1261 caactcgact ccatgaagtc ggaatcgcta gtaatcgtag atcagaatgc tacggtgaat

1321 acgttcccgg gccttgtaca caccgcccgt cacaccatgg gagtgggttg caaaagaagt

1381 aggtagctta accttcggga gggcgc

//

OK504397 <https://www.ncbi.nlm.nih.gov/nuccore/OK504397>

**Escherichia coli strain X23sq2 16S ribosomal RNA gene, partial sequence**

1 tgcagtcgaa cggtaacaga aagcagcttg ctgctttgct gacgagtggc ggacgggtga

61 gtaatgtctg ggaaactgcc tgatggaggg ggataactac tggaaacggt agctaatacc

121 gcataacgtc gcaagaccaa agagggggac cttcgggcct cttgccatcg gatgtgccca

181 gatgggatta gcttgttggt ggggtaacgg ctcaccaagg cgacgatccc tagctggtct

241 gagaggatga ccagccacac tggaactgag acacggtcca gactcctacg ggaggcagca

301 gtggggaata ttgcacaatg ggcgcaagcc tgatgcagcc atgccgcgtg tatgaagaag

361 gccttcgggt tgtaaagtac tttcagcggg gaggaagggg agtaaagtta atacctttgc

421 tcattgacgt tacccgcaga agaagcaccg gctaactccg tgccagcagc cgcggtaata

481 cggagggtgc aagcgttaat cggaattact gggcgtaaag cgcacgcagg cggtttgtta

541 agtcagatgt gaaatccccg ggctcaacct gggaactgca tctgatactg gcaagcttga

601 gtctcgtaga ggggggtaga attccaggtg tagcggtgaa atgcgtagag atctggagga

661 ataccggtgg cgaaggcggc cccctggacg aagactgacg ctcaggtgcg aaagcgtggg

721 gagcaaacag gattagatac cctggtagtc cacgccgtaa acgatgtcga cttggaggtt

781 gtgcccttga ggcgtggctt ccggagctaa cgcgttaagt cgaccgcctg gggagtacgg

841 ccgcaaggtt aaaactcaaa tgaattgacg ggggcccgca caagcggtgg agcatgtggt

901 ttaattcgat gcaacgcgaa gaaccttacc tggtcttgac atccacggaa gttttcagag

961 atgagaatgt gccttcggga accgtgagac aggtgctgca tggctgtcgt cagctcgtgt

1021 tgtgaaatgt tgggttaagt cccgcaacga gcgcaaccct tatcctttgt tgccagcggt

1081 ccggccggga actcaaagga gactgccagt gataaactgg aggaaggtgg ggatgacgtc

1141 aagtcatcat ggcccttacg accagggcta cacacgtgct acaatggcgc atacaaagag

1201 aagcgacctc gcgagagcaa gcggacctca taaagtgcgt cgtagtccgg attggagtct

1261 gcaactcgac tccatgaagt cggaatcgct agtaatcgtg gatcagaatg ccacggtgaa

1321 tacgttcccg ggccttgtac acaccgcccg tcacaccatg ggagtgggtt gcaaaagaag

1381 taggtagctt aaccttcggg agggcgcta

OK504398 <https://www.ncbi.nlm.nih.gov/nuccore/OK504398>

**Klebsiella pneumoniae strain X26sq2 16S ribosomal RNA gene, partial sequence**

1 tgcagtcgag cggtagcaca gagagcttgc tctcgggtga cgagcggcgg acgggtgagt

61 aatgtctggg aaactgcctg atggaggggg ataactactg gaaacggtag ctaataccgc

121 ataatgtcgc aagaccaaag tgggggacct tcgggcctca tgccatcaga tgtgcccaga

181 tgggattagc tagtaggtgg ggtaacggct cacctaggcg acgatcccta gctggtctga

241 gaggatgacc agccacactg gaactgagac acggtccaga ctcctacggg aggcagcagt

301 ggggaatatt gcacaatggg cgcaagcctg atgcagccat gccgcgtgta tgaagaaggc

361 cttcgggttg taaagtactt tcagcgggga ggaaggcgat aaggttaata accttgtcga

421 ttgacgttac ccgcagaaga agcaccggct aactccgtgc cagcagccgc ggtaatacgg

481 agggtgcaag cgttaatcgg aattactggg cgtaaagcgc acgcaggcgg tctgtcaagt

541 cggatgtgaa atccccgggc tcaacctggg aactgcattc gaaactggca ggctagagtc

601 ttgtagaggg ggggtagaat tccaggtgta gcggtgaaat gcgtagagat ctggaggaat

661 accggtggcg aaggcggccc cctggacaaa gactgacgct caggtgcgaa agcgtgggga

721 gcaaacagga ttagataccc tggtagtcca cgccgtaaac gatgtcgatt tgggaggttg

781 tgcccttgag gcgtggcttc cggagctaac gcgttaaatc gaccgcctgg gggagtacgg

841 ccgcaaggtt aaaactcaaa tgaattgacg ggggcccgca caagcggtgg agccatgtgg

901 tttaattcga tgcaacgcga agaaccttac ctggtcttga catccacaga acttagcaga

961 gatgctttgg tgccttcggg aactgtgaga caggtgctgc atggctgtcg tcagctcgtg

1021 ttgtgaaatg tttgggttaa gtcccgcaac gagcgcaacc cttatccttt gttgccagcg

1081 gtccggccgg gaactcaaag gagactgcca gtgataaact ggaggaaggt ggggatgacg

1141 tcaagtcatc atggccctta cgaccagggc tacacacgtg ctacaatggc atatacaaag

1201 agaagcgacc tcgcgagagc aagcggacct cataaagtat gtcgtagtcc ggattggagt

1261 ctgcaactcg actccatgaa gtcggaatcg ctagtaatcg tagatcagaa tgctacggtg

1321 aatacgttcc cgggccttgt acacaccgcc cgtcacacca tgggagtggg ttgcaaaaga

1381 agtaggtagc ttaaccttcg ggagggcgct a

OK504399 <https://www.ncbi.nlm.nih.gov/nuccore/OK504399>

**Enterococcus faecium strain X8sq2 16S ribosomal RNA gene, partial sequence**

1 tgcagtcgaa cgcttctttt tcaccggagc ttgctccacc ggaaaaagag gagtggcgaa

61 cgggtgagta acacgtgggt aacctgccca tcagaagggg ataacacttg gaaacaggtg

121 ctaataccgt ataacaatcg aaaccgcatg gttttgattt gaaaggcgct ttcgggtgtc

181 gctgatggat ggacccgcgg tgcattagct agttggtgag gtaacggctc accaaggcca

241 cgatgcatag ccgacctgag agggtgatcg gccacattgg gactgagaca cggcccaaac

301 tcctacggga ggcagcagta gggaatcttc ggcaatggac gaaagtctga ccgagcaacg

361 ccgcgtgagt gaagaaggtt ttcggatcgt aaaactctgt tgttagagaa gaacaaggat

421 gagagtaact gttcatccct tgacggtatc taaccagaaa gccacggcta actacgtgcc

481 agcagccgcg gtaatacgta ggtggcaagc gttgtccgga tttattgggc gtaaagcgag

541 cgcaggcggt ttcttaagtc tgatgtgaaa gcccccggct caaccgggga gggtcattgg

601 aaactgggag acttgagtgc agaagaggag agtggaattc catgtgtagc ggtgaaatgc

661 gtagatatat ggaggaacac cagtggcgaa ggcggctctc tggtctgtaa ctgacgctga

721 ggctcgaaag cgtggggagc aaacaggatt agataccctg gtagtccacg ccgtaaacga

781 tgagtgctaa gtgttggagg gtttccgccc ttcagtgctg cagctaacgc attaagcact

841 ccgcctgggg agtacgaccg caaggttgaa actcaaagga attgacgggg gcccgcacaa

901 gcggtggagc atgtggttta attcgaagca acgcgaagaa ccttaccagg tcttgacatc

961 ctttgaccac tctagagata gagcttcccc ttcgggggca aagtgacagg tggtgcatgg

1021 ttgtcgtcag ctcgtgtcgt gagatgttgg gttaagtccc gcaacgagcg caacccttat

1081 tgttagttgc catcattcag ttgggcactc tagcaagact gccggtgaca aaccggagga

1141 aggtggggat gacgtcaaat catcatgccc cttatgacct gggctacaca cgtgctacaa

1201 tgggaagtac aacgagtcgc gaagtcgcga ggctaagcta atctcttaaa gcttctctca

1261 gttcggattg caggctgcaa ctcgcctgca tgaagccgga atcgctagta atcgcggatc

1321 agcacgccgc ggtgaatacg ttcccgggcc ttgtacacac cgcccgtcac accacgagag

1381 tttgtaacac ccgaagtcgg tgaggtaacc tttggagcca gccg
